# Editorial: Focus feature on biomarkers in network neuroscience

**DOI:** 10.1162/netn_e_00249

**Published:** 2022-06-01

**Authors:** Linda Douw, Mario Senden, Martijn van den Heuvel

**Affiliations:** Department of Anatomy and Neurosciences, Amsterdam Neuroscience, UMC, Vrije Universiteit Amsterdam, Amsterdam, Netherlands; Department of Cognitive Neuroscience, Faculty of Psychology and Neuroscience, Maastricht University, Maastricht, Netherlands; Maastricht Brain Imaging Centre, Faculty of Psychology and Neuroscience, Maastricht University, Maastricht, Netherlands; Department of Complex Trait Genetics, Center for Neurogenomics and Cognitive Research, Vrije Universiteit Amsterdam, Amsterdam Neuroscience, Amsterdam, Netherlands; Department of Child Psychiatry, Amsterdam UMC, Vrije Universiteit Amsterdam, Amsterdam, Netherlands

**Keywords:** Clinical neuroscience, Neurology, Psychiatry, Graph theory, Connectome, Treatment monitoring, Response prediction, Personalized medicine, Precision medicine

## Abstract

There is an ongoing need for novel biomarkers in clinical neuroscience, as diagnosis of neurological and psychiatric disorders is hampered by the pronounced overlap of behavioral symptoms and other pathophysiological characteristics. The question that this Focus Feature puts center stage is whether network-based biomarkers may provide a viable tool for distinguishing between disordered populations or whether they may yield only limited differentiating power because of largely shared network characteristics across conditions.

Multiple approaches exist to address the question of whether network-based biomarkers may have clinical utility; we explore many of these in this Focus Feature. Blomsma and colleagues (Blomsma et al., 2022) focus on the minimum spanning tree (MST) as a tool that potentially unearths differences between neurological or psychiatric pathologies and healthy controls. The authors propose that the MST offers a theoretical approach towards thresholding adjacency matrices, by including the subset of edges that maximize connectivity while not forming any loops or cycles. They perform a systematic review on MST studies using different network sizes and assess disease specificity and transdiagnostic sensitivity of MST-based metrics. Although contradictions were present in results across studies, several trends could nevertheless be distilled within and across disease categories. Interestingly, it became clear that the MST measure of leaf fraction depends on network size independent of disease population, limiting the interpretability of this measure across studies with different methodologies. The authors also suggest that reporting guidelines are needed in network neuroscience, emphasizing the importance of always reporting numeric values for network metrics as well as the analysis tools used as completely as possible.

The work by Rodriguez-Cruces and colleagues (Rodriguez-Cruces et al., 2022) uses a different strategy towards biomarker exploration: They reviewed the current state of network neuroscience literature in three particular epilepsy syndromes that are difficult to differentiate, with the hope to identify shared as well as syndrome-specific network substrates. Indeed, the authors conclude that “brain network measures may ultimately serve as powerful intermediary phenotypes to study effects of biological as well as environmental factors on cognitive systems in epileptic patients, including medication effects, disease status, and baseline genetic factors,” indicating that there are indeed common and specific network markers for these syndromes. They recommend future work to incorporate not only multimodal imaging, but also genetic testing as well as rigorous clinical phenotyping. Together with multisite data collection, the authors posit that such work could pave the way towards incorporation of network-based biomarkers in clinical practice in epilepsy.

This Focus Feature also provides and evaluates new network-based biomarkers. Kulik and colleagues (Kulik et al., 2022) explore associations between structural and functional connectivity in a cohort of multiple sclerosis (MS) patients, and investigate whether (alterations in) this relation could be a useful biomarker for cognitive impairment in MS. Although there were significant differences in structure-function coupling between cognitively impaired patients and matched healthy controls, receiver operating characteristic (ROC) curves revealed that these group-level differences did not significantly differentiate between groups. This result underlines the importance of reporting classification accuracy for potential biomarkers, particularly in the context of significant group differences.

Iraji and colleagues (Iraji et al., 2022) use the newly defined method of multiscale independent component analysis (msICA) to study shared and specific connectivity patterns across males and females in schizophrenia. This method allows for data-driven natural detection of functional sources across different spatial scales. Using three large cohorts, the authors report on shared network differences between male and female schizophrenic patients, but also reveal sex-specific effects that correlated with symptom scores. The authors emphasize the importance of carefully incorporating sex in the development of diagnostic, predictive, and/or monitoring biomarkers in schizophrenia.

Finally, Scheijbeler and colleagues (Scheijbeler et al., 2022) introduce a network version of permutation entropy as a novel biomarker for early-stage Alzheimer’s disease (AD). This measure integrates information on local signal variability and complexity with nonlinear coupling. They indeed find group differences between patients with early AD and a control population. Moreover, classification accuracy through ROC curve analysis was comparable to the current state-of-the-art biomarker in this context, namely relative theta power. Future work may aim to replicate this finding in larger samples.

There is promise for network-based biomarkers in clinical neuroscience. An important step towards adequate investigation of brain network measures as biomarkers is to consistently report reliability, reproducibility, sensitivity, and specificity of such measures. The increasing availability of large databases with multimodal data in order to crosslink different approaches will aid development of biomarkers suitable for differential diagnosis and prognosis. We hope that the work included in this Focus Feature may inform future studies into this important topic.
